# Supplementary material for: Causal Relationship between Aging and Anorexia Nervosa: A White-Matter-Microstructure-Mediated Mendelian Randomization Analysis
Source: Biomedicines. 2024 Aug 16;12(8):1874. doi: 10.3390/biomedicines12081874 (PMC11351342; doi:10.3390/biomedicines12081874)
Supplement: Supplementary file 1 [file biomedicines-12-01874-s001.zip › Supplementary Figure/Figure S4/Singe SNP plot of MR analysis for TL to ubm-b-1518.pdf]

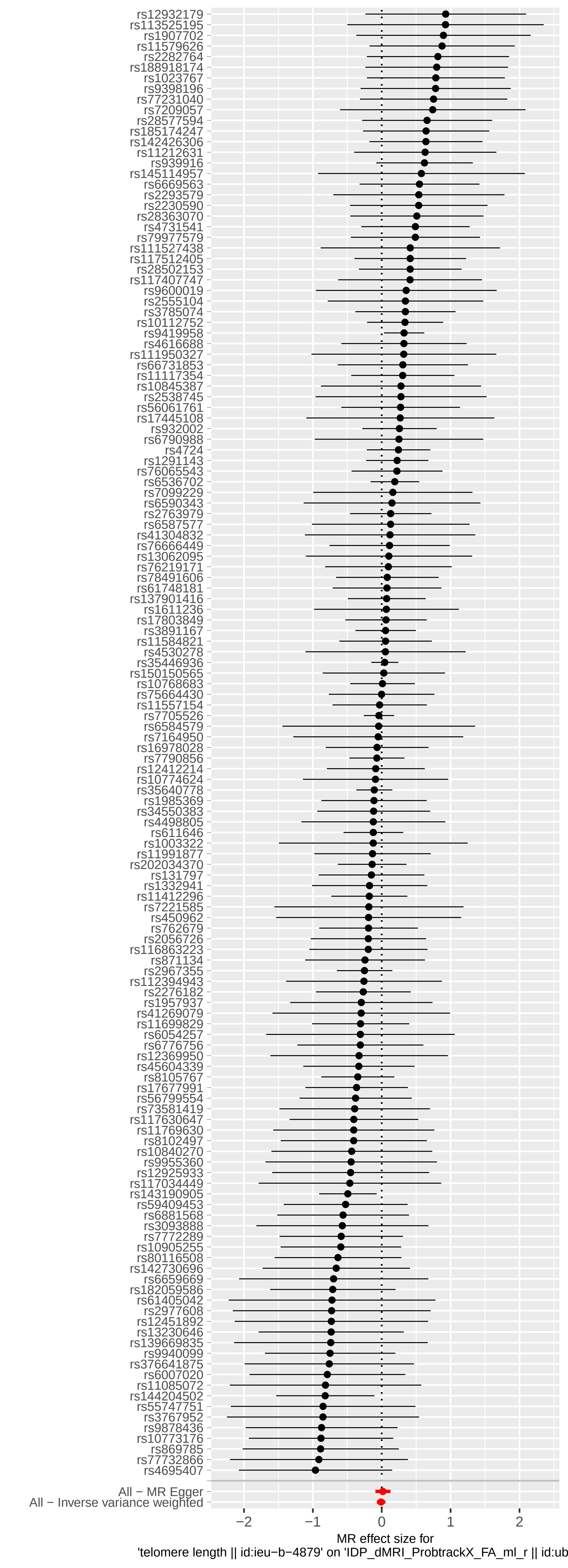
rs12932179
rs113525195
rs1907702
rs11579626
rs2282764
rs188918174
rs1023767
rs9398196
rs77231040
rs7209057
rs28577594
rs185174247
rs142426306
rs11212631
rs939916
rs145114957
rs6669563
rs2293579
rs2230590
rs28363070
rs4731541
rs79977579
rs111527438
rs117512405
rs28502153
rs117407747
rs9600019
rs2555104
rs3785074
rs10112752
rs9419958
rs4616688
rs111950327
rs66731853
rs11117354
rs10845387
rs2538745
rs56061761
rs17445108
rs932002
rs6790988
rs4724
rs1291143
rs76065543
rs6536702
rs7099229
rs6590343
rs2763979
rs6587577
rs41304832
rs76666449
rs13062095
rs76219171
rs78491606
rs61748181
rs137901416
rs1611236
rs17803849
rs3891167
rs11584821
rs4530278
rs35446936
rs150150565
rs10768683
rs75664430
rs11557154
rs7705526
rs6584579
rs7164950
rs16978028
rs7790856
rs12412214
rs10774624
rs35640778
rs1985369
rs34550383
rs4498805
rs611646
rs1003322
rs11991877
rs202034370
rs131797
rs1332941
rs11412296
rs7221585
rs450962
rs762679
rs2056726
rs116863223
rs871134
rs2967355
rs112394943
rs2276182
rs1957937
rs41269079
rs11699829
rs6054257
rs6776756
rs12369950
rs45604339
rs8105767
rs17677991
rs56799554
rs73581419
rs117630647
rs11769630
rs8102497
rs10840270
rs9955360
rs12925933
rs117034449
rs143190905
rs59409453
rs6881568
rs3093888
rs7772289
rs10905255
rs80116508
rs142730696
rs6659669
rs182059586
rs61405042
rs2977608
rs12451892
rs13230646
rs139669835
rs9940099
rs376641875
rs6007020
rs11085072
rs144204502
rs55747751
rs3767952
rs9878436
rs10773176
rs869785
rs77732866
rs4695407
All − MR Egger
All − Inverse variance weighted
−2
−1
0
1
2
MR effect size for
'telomere length || id:ieu−b−4879' on 'IDP_dMRI_ProbtrackX_FA_ml_r || id:ub
